# The Psychological Emptiness Scale: a psychometric evaluation

**DOI:** 10.1192/bjo.2023.649

**Published:** 2024-02-01

**Authors:** Shona Joyce Herron, Rob Saunders, Fabio Sani, Janet Feigenbaum

**Affiliations:** Acute Mental Health Services, Central and North West London NHS Foundation Trust, UK; and Department of Clinical, Educational and Health Psychology, University College London, UK; CORE Data Lab, Department of Clinical, Educational and Health Psychology, University College London, UK; Division of Psychology, University of Dundee, UK; Department of Clinical, Educational and Health Psychology, University College London, UK

**Keywords:** Emptiness, personality disorders, rating scales, suicide, clinical outcomes measures

## Abstract

**Background:**

Feelings of emptiness are commonly reported as deeply distressing experiences. Despite established relationships between emptiness and many mental health difficulties, alongside self-harm and suicide, further study into this phenomenon has been restricted by vague definition and clinical measures with limited utility. Recently the first definition validated by individuals with lived experience of emptiness has been conceptualised, providing an opportunity to create a new measure of emptiness.

**Aims:**

This study aimed to psychometrically evaluate the 31-item Psychological Emptiness Scale (PES), identifying redundancy, and thus creating a psychometrically robust scale with optimised clinical utility.

**Method:**

Utilising an online survey design, 768 participants completed the 31 items of the initial PES alongside other measures of mental health. Exploratory factor analysis was conducted, and item response theory employed to identify item redundancy and reduce test burden. Expert clinicians provided ratings of each item's clinical relevance and, combined with the psychometric analysis, led to the removal of a number of items. Confirmatory factor analysis was then undertaken. Reliability including test–retest, validity and sensitivity of the measure were evaluated.

**Results:**

A two-factor structure encompassing ‘nothingness’ and ‘detachment’ was identified, and found to have acceptable fit. The resulting 19-item PES was found to have internal consistency (*α* = 0.95), convergent validity and test–retest reliability.

**Conclusions:**

This study demonstrated strong psychometric properties of the PES. The PES has potential to support research into the role of emptiness in psychological distress and treatment in clinical practice.

Emptiness is a baffling, persistent and deeply distressing psychological experience.^1,2^ Although there is ample evidence for emptiness to be relatively common and transdiagnostic,^3,4^ current literature fails to consider it outside the conceptualisation of emptiness as a diagnostic feature of borderline personality disorder (BPD).^5^ However, more recent work by Herron and Sani has created a validated definition of emptiness, identifying it to be an all-encompassing, distressing and transdiagnostic experience akin to an existential feeling.^4^

**‘**[Emptiness is] A feeling that one is going through life mechanically, devoid of emotions and purpose, and therefore is empty inside, with emptiness often being bodily felt in the form of a discomfort in the chest. This is coupled with feelings that one is disconnected from others, in some way invisible to others, and unable to contribute to a world that remains the same, but from which one is distant and detached.’^4^

## Measuring emptiness

Previous narrow conceptualisations have impeded real progress in the construction of valid assessment tools. In line with the idea that emptiness is a mere ‘symptom’ of BPD, researchers have often assessed it using single items taken from measures of BPD,^6^ inevitably ignoring the inherent phenomenological complexity of this experience. Even when researchers have tried to develop more nuanced multi-item instruments, scale validation has focussed almost exclusively on people with a diagnosis of BPD. This limitation applies, for instance, to the Experienced Levels of Emptiness Scale,^7^ the Emptiness Scale^8^ and the Sense of Emptiness Scale.^9^

Recognising these challenges, Price and colleagues^10^ developed a self-report measure of emptiness aiming to address it as a transdiagnostic experience. The resulting five-item Subjective Emptiness Scale displayed good internal consistency and correlated with internalising difficulties, as well as self-harm, suicidality and substance use. However, items were based on online content posted by people with a psychiatric diagnosis and on transcripts of interviews with people specifically diagnosed with BPD. Again, centring this diagnosis is not entirely in line with the evidence that emptiness is a transdiagnostic experience. Additionally, this work set out to create a unidimensional measure of emptiness, presuming a single factor of emptiness. Thereby, this scale continues to be based on theoretical assumptions that are not necessarily justified. Additionally, all of the aforementioned scales were developed before the existence of a definition of the experience, and thus before the phenomenology of this experience elucidated.

Thus, we have developed the Psychological Emptiness Scale (PES), using a set of items not predominantly based on reports given by people with BPD, and that do not presuppose a single-factor structure. These items are derived from the findings of a phenomenological study conducted by Herron and Sani,^4^ which involved a sample of individuals either with or without any mental health diagnoses, of which only a minority had been diagnosed with BPD. The personal accounts of emptiness produced by respondents were used to describe the prototypical presentation of emptiness, which highlighted a self that is experienced as numbed, devitalised and disconnected from other people and from an impersonal world that is devoid of significance. Importantly, within this work, this definition was submitted to an independent sample of people with lived experience of emptiness for qualitative evaluation, confirming the high level of accuracy of the description to their lived experience. This served the further aim of providing adequate pre-testing for the PES because they were directly taken from the validated definition and participant responses.

## The current study

The aim of this study is to provide a robust psychometric evaluation of the PES, which includes factor analytic enquiry, and the use of item response theory modelling to identify potential redundancy. The resulting scale will then be subject to further reliability and validity testing.

## Method

### Participants

Participants were recruited with an online questionnaire that was advertised on social media, institutional websites and online pages related to mental health, over a 16-week period. A study website was created (https://ucjush9.wixsite.com/emptiness) and was launched on 20 April 2021, following receipt of ethical approval. Inclusion criteria were: being over 18 years of age and self-identifying as having ever felt empty. Participants were excluded if they self-reported a diagnosis of severe mental illness with psychotic features (schizophrenia, bipolar disorder or schizoaffective disorder), were unable to read English fluently or reported having never felt empty. Participants were first asked whether they had ever felt empty and were provided with the definition of emptiness from Herron and Sani,^4^ defining emptiness to be a psychologically felt experience. All whom answered yes, were then asked to provide informed written consent to take part. Surveys were considered incomplete if participants had not proceeded through all questions to the final webpage, and was interpreted as intention to withdraw from the study.

Ethical approval was granted by the University College London Research Ethics Committee on 14 April 2021 (ethics number: 19415/001).

### Measures

The first part of the survey asked about age, gender, ethnicity, country of residence and primary occupation. Participants were then asked whether they had ever had thoughts of or attempted suicide, and whether they had ever self-harmed. For each, individuals could answer yes, no or prefer not to say. They were then asked to disclose whether they had ever received a diagnosis of a personality disorder. Participants then completed the measures described below, which were chosen because they were brief, thoroughly validated measures encompassing internal psychological distress, relational experiences and engagement with the world, capturing the three components of emptiness.^4^ Thus, scores on these measures could be expected to produce convergent results to an emptiness scale that appropriately captured the full experience of the experience.

#### The PES

The PES is a 31-item measure created by the authors S.J.H. and F.S., wherein all items were generated from the validated definition of emptiness.^4^ Items were generated by closely attending to the definition, ensuring that the three conceptual domains and nine components identified in the initial study were accounted for within the items. The measure asks, ‘Please state how often you have had the following experiences over the last month’, asking participants to then rate items on a four-point scale. A month was chosen as the scale duration because of the persistence and chronicity of emptiness in previous work,^2,6^ and evidence linking greater chronicity to greater distress.^4^ Items on the scale included ‘never’, ‘sometimes’, ‘often’ and ‘all of the time’. Development of items aimed to use simple and jargon-free language, and effort was made to retain the wordings used by individuals with lived experience. The validity and reliability of this measure is not yet known, the evaluation of which is the purpose of this work.

#### The Clinical Outcomes in Routine Evaluation 10

The Clinical Outcomes in Routine Evaluation 10 (CORE-10) is a ten-item self-report measure, used routinely in clinical practice to assess psychological distress, which has been validated for use with adults in clinical and general population. The internal consistency of the CORE-10 was found to be *α* = 0.90.^11^

#### The Standardised Assessment of Personality: Abbreviated Scale

The Standardised Assessment of Personality: Abbreviated Scale (SAPAS) is an eight-item self-report measure, used to screen for personality disorders in routine psychiatric assessment.^12^ In the general population, a score of 4 was found to have moderate accuracy in identifying a potential personality disorder, thus this was the cut-off used for the purpose of this study.^13^

#### The Revised UCLA Loneliness Scale

The Revised UCLA Loneliness Scale is a measure of loneliness with high internal consistency (*α* = 0.94) and good test–retest reliability (*r* = 0.73), and correlated with qualitative self-reports of loneliness.^14–16^

#### The Satisfaction with Life Scale

The Satisfaction with Life Scale (SWLS) is a self-report measure of life satisfaction^17^ that correlates with measures of mental health^18^ and is predictive of future suicide attempts. It has been shown to have good internal consistency (*α* = 0.85) and construct validity.^17–19^

### Procedure

The survey was created in and hosted on Qualtrics software for Windows, version 04/2021 (Qualtrics, UT, USA; see https://www.qualtrics.com). Data collection at time point 1 began on 20 April 2021 and ended on 4 August 2021. Participants at time point 1 were asked to provide an email address and generate a unique identifier to allow for data pairing, if they would like to take part in a further round of data collection that focused on the test–retest reliability of the PES. Those who agreed were emailed on 27 August 2021, with data collection ending on 23 September 2021.

### Clinician group participants and recruitment

To assess face validity of the PES items, clinicians from a specialist personality disorder service in London (led by author J.F.) were surveyed. Clinicians rated how important they viewed each of the 31 items in helping to understand emptiness, from ‘not at all important’ to ‘extremely important’, on a five-point scale. Responses were used to determine the clinical utility and face validity of the measure, with ratings considered alongside psychometric evaluation data to inform decisions about item redundancy.

### Statistical analysis

Floor and ceiling effects were assessed by calculating the percentage of the sample who scored the maximum or minimum score on each PES item (Supplementary Appendix 1 available at https://doi.org/10.1192/bjo.2023.649).

For analyses, two groups were created from the full sample: a high personality disorder group and a low personality disorder group, using SAPAS scores ≥4^13^ as the cut-off for the high personality disorder group. Although this does not constitute a confirmed formal diagnosis of a personality disorder, it allowed us to identify a group who were likely to display greater personality disorder traits.

Demographics were calculated for the full sample, as well as the low and high personality disorder groups separately. Two-sample *t*-tests, chi-squared tests and Fisher's exact tests were conducted to explore potential differences between the high and low personality disorder groups at time point 1 on the demographic and mental health variables. These were repeated to identify any significant differences between the time point 1 and time point 2 sample demographics. Some categories were collapsed because of small, expected cell frequencies: within country of residence, ‘UK’ and ‘Europe’ were collapsed to create one overall European group; and within occupation unemployed, ‘unemployed due to health or disability’ and ‘unemployed due to COVID-19’ were collapsed to create one unemployed group. Because of the non-normal distribution of the PES scores, a Mann–Whitney *U*-test was also undertaken to examine total emptiness scores between people with and without high personality disorder scores.

To provide a robust psychometric evaluation of the PES, the included sample was randomly split 50/50 stratified by personality disorder status, with the first half as the exploratory sample and the second half the confirmatory sample. The psychometric analyses were conducted in three phases, detailed below.

#### Phase 1: exploratory sample

Exploratory factor analysis (EFA) was first conducted on the exploratory sample to identify potential subfactors of the 31-item PES. Varimax rotation was employed, and the identification of relevant factors was informed by eigenvalues over 1. Once the optimal factor solution was chosen, potential item redundancy was informed through item response theory (IRT). A graded response model was estimated on each identified factor, with item performance indicated by item information functions (IIFs) and item characteristic curves. These provide information about how items relate to the latent trait, thus identifying those with a large degree of overlap and therefore candidates for removal.^20^ Those providing information <1 were removed, with retained items then becoming the revised PES and subject for further evaluation described below.

In addition to the psychometric analyses, the face validity of the revised PES was established through consultation with clinicians who had experience working with emptiness. Clinicians completed a questionnaire asking about their experience of working with clients expressing emptiness and rated the items on the PES in relation to their importance for measuring emptiness. Mean clinician ratings for each item were calculated and used to assist in the decision-making process regarding pruning of the measure.

#### Phase 2: confirmatory sample

Once the revised and shortened PES had been established, analyses were conducted with the confirmatory sample on the retained PES items, to assess the revised measure with a sample independent of that used in the initial analyses. Confirmatory factor analysis (CFA) was first conducted on the newly proposed factor structure of the PES. The root mean square error of approximation, comparative fit index (CFI) and standardised root mean square residual were used to assess model fit. Root mean square error of approximation and standardised root mean square residual values below 0.8, and CFI values above 0.95, were taken to indicate acceptable fit, in line with standards in the literature.^21^

#### Phase 3: full sample

##### Reliability

The internal consistency of the revised PES was assessed with Cronbach's alpha alongside bias-corrected and accelerated bootstrap confidence interval (95% BCa CI) to adjust for skewness, combining the exploratory and confirmatory samples (full sample). Test–retest reliability was assessed through Spearman's Rho between time point 1 and 2 PES scores.

##### Validity

Tests of normality were significant (*P* < 0.01), and thus Spearman's Rho was used to assess convergent validity between total emptiness scores, factor scores and the other measures of well-being: psychological distress, satisfaction with life, loneliness and personality disorder traits. Analyses were carried out with the full sample, as well as separately for the high and low personality disorder groups.

For this reason, Spearman's Rho was also used to assess relationships between total emptiness scores, as well as the scores on the two factors, in relation to self-harm, suicidal thoughts and attempted suicide. For these analyses, those who answered prefer not to say were excluded.

##### Power

Based on Consensus-Based Standards for the Selection of Health Measurement Instruments (COSMIN) guidance, to perform all intended analysis, the number of participants per item was calculated, with a minimum sample size of 465 participants required to meet all stipulations.^22^

IRT was undertaken with StataMP for Windows version 17.0, with all other analyses conducted with JASP for Windows (JASP Team, University of Amsterdam, The Netherlands; see https://jasp-stats.org/) version 0.16.1.0 or SPSS for Windows version 26.

## Results

### Participants

**Fig. 1:**
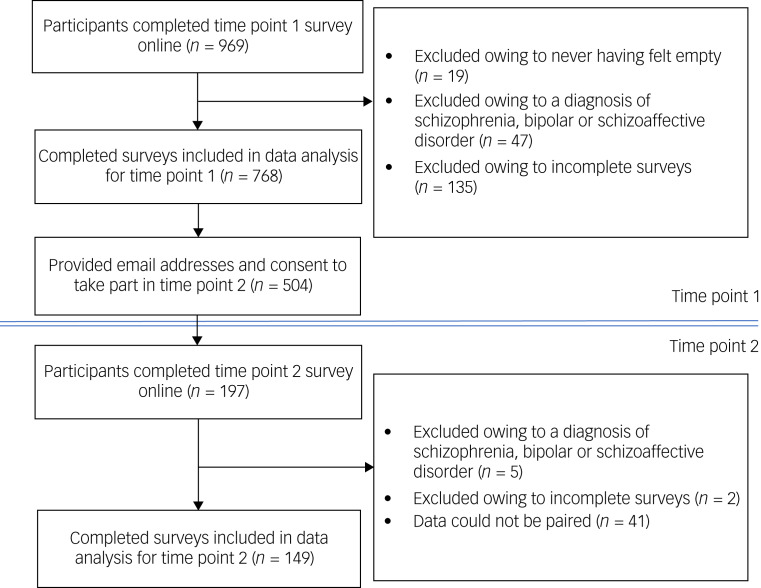
Consolidated Standards of Reporting Trials (CONSORT) diagram showing participant recruitment for time points 1 and 2.

Of the 969 individuals who took part, 201 individuals were removed (see Fig. 1). This resulted in 768 completed surveys from individuals who self-reported ever feeling empty. Of the 768 completed surveys, 504 (65.6%) gave written consent to be contacted again, of which 197 participants took part in the second survey assessing test–retest reliability. Upon inspection of the data, 48 participants were removed (see Fig. 1 for exclusions) resulting in a total of 149 completed surveys used in the retest analysis.

Demographics (Table 1) and mental health demographics (Table 2) were calculated for the full sample at time point 1, as well as separately for the high and low personality disorder groups.

**Table 1:** Descriptive statistics for the full sample, high personality disorder group and low personality disorder group at time point 1

	Full sample (*N* = 768)	High personality disorder group (*n* = 490)	Low personality disorder group (*n* = 278)
	*n* (%)	*n* (%)	*n* (%)
Gender			
Male	234 (30.9)	166 (33.9)	68 (24.5)
Female	489 (63.5)	287 (58.6)	202 (72.7)
Non-binary	24 (3.1)	19 (3.9)	5 (1.8)
Transman	7 (0.9)	6 (1.2)	1 (0.4)
Transwoman	6 (0.8)	5 (1.0)	1 (0.4)
Other	8 (1.0)	7 (1.4)	1 (0.4)
Age, years			
Mean (s.d.)	29.6 (9.7)	28.3 (8.8)	31.8 (11.0)
Median	27.0	26	28
Range	18–68	18–67	18–68
Relationship status			
Co-habiting	82 (10.6)	45 (9.2)	37 (13.3)
Divorced/separated	18 (2.3)	9 (1.8)	9 (3.2)
In a relationship	159 (20.7)	86 (17.6)	73 (26.5)
Married	110 (14.3)	67 (13.7)	43 (15.5)
Single	388 (50.2)	275 (56.1)	112 (40.3)
Widowed	3 (0.4)	1 (0.2)	2 (0.7)
Other	9 (1.2)	7 (1.4)	2 (0.7)
Employment status			
Full-time employed	370 (48.1)	206 (42.0)	164 (59.0)
Home maker	8 (1.0)	8 (1.6)	0
Part-time employed	72 (9.4)	47 (9.6)	24 (8.6)
Retired	5 (0.7)	2 (0.4)	3 (1.1)
Student	206 (26.8)	136 (27.8)	70 (25.2)
Unemployed	48 (6.3)	41 (8.4)	7 (2.5)
Unemployed owing to COVID-19	8 (1.0)	7 (1.4)	1 (0.4)
Unemployed owing to health or disability	52 (6.8)	43 (8.8)	9 (3.2)
Ethnicity			
Any White background	631 (82.2)	403 (82.2)	233 (83.8)
Asian or British Asian	52 (6.7)	29 (5.9)	23 (8.3)
Mixed or multiple ethnic groups	28 (3.6)	19 (3.9)	9 (3.2)
Black, African, Caribbean or Black British	12 (1.6)	9 (1.8)	3 (1.0)
Other ethnic groups	26 (3.4)	21 (4.3)	5 (1.8)
Did not disclose	19 (2.5)	9 (1.8)	5 (1.8)
Area of residence			
UK (Scotland, England, Wales and Northern Ireland)	433 (56.0)	226 (46.1)	207 (74.5)
Europe (including Ireland)	104 (13.5)	77 (15.7)	27 (9.7)
North America, Central America and Canada	165 (21.3)	136 (27.8)	29 (10.4)
South America	9 (1.2)	9 (1.8)	0 (0.0)
Middle East	2 (0.4)	1 (0.2)	1 (0.4)
Asia	24 (3.1)	17 (3.5)	7 (2.5)
Africa	6 (0.8)	5 (1.0)	1 (0.4)
Australasia	17 (2.2)	12 (2.4)	5 (1.8)
Did not disclose	11 (1.4)	5 (1.0)	6 (2.2)
Average time taken to complete survey	2033.9 secs/33.9 mins	2667.0 secs/44.5 mins	945.9 secs/15.8 mins

**Table 2:** Mental health descriptive statistics for full sample, high personality disorder group and low personality disorder group at time point 1

	Full sample (*N* = 768)	High personality disorder group (*n* = 490)	Low personality disorder group (*n* = 278)
	*n* (%)		
Ever felt empty			
All of the time	124 (16.0)	109 (22.2)	14 (5.0)
Often	348 (44.9)	248 (50.6)	97 (34.9)
Sometimes	231 (29.8)	120 (24.5)	111 (39.9)
Rarely	69 (8.9)	13 (2.7)	56 (20.1)
Self-harm (4 with missing data)			
Yes	412 (53.2)	306 (62.4)	106 (38.1)
No	350 (45.2)	179 (36.5)	171 (61.5)
Prefer not to say	6 (0.8)	5 (1.0)	1 (0.4)
Thoughts of suicide (4 with missing data)			
Yes	587 (75.7)	420 (85.7)	167 (60.1)
No	165 (21.3)	62 (12.7)	103 (37.1)
Prefer not to say	16 (2.1)	8 (1.6)	8 (2.9)
Attempted suicide (4 with missing data)			
Yes	179 (23.1)	145 (29.6)	34 (12.2)
No	575 (74.2)	332 (67.8)	243 (87.4)
Prefer not to say	14 (1.8)	13 (2.7)	1 (0.4)
Personality disorder diagnoses			
None	622 (80.6)	357 (72.9)	261 (93.9)
Number who self-reported diagnosis of at least one personality disorder	149 (19.4)	133 (27.1)	17 (6.1)
Number who scored ≥4 on SAPAS	490 (63.7)	490 (100)	0
Frequency of personality disorder diagnoses among those who had received at least one formal personality disorder diagnosis^a^	(*n* = 150)	(*n* = 133)	(*n* = 17)
Personality disorder type	Frequencies		
Borderline	85	75	10
Emotionally unstable	30	25	5
Obsessive–compulsive	12	12	0
Schizotypal	4	4	0
Schizoid	29	25	4
Antisocial	11	17	0
Narcissistic	2	2	0
Avoidant	19	17	2
Dependant	3	2	1
Histrionic	1	1	0
Paranoid	4	3	1
Range number of personality disorder diagnoses received	1–4	1–4	1–2

SAPAS, Standardised Assessment of Personality: Abbreviated Scale.

a. Many individuals reported having received multiple personality disorder diagnoses.

Comparative analysis between the high and low personality disorder groups identified significant differences regarding age (*t*(765) = 4.83, *P* < 0.001), gender (*χ^2^*(5, *N* = 768) = 17.84, *P* = 0.004), employment (Fisher's exact test *P* < 0.001), relationship status (Fisher's exact test *P* < 0.001), country of residence (Fisher's exact test *P* < 0.001) and whether participants had received a formal diagnosis of a personality disorder (*χ^2^*(1, *N* = 768) = 49.90, *P* < 0.001). Group differences regarding ethnicity were non-significant, as was the difference between time taken to complete the survey. Between the high and low personality disorder groups, there was also a significant difference in presence of suicidal thoughts (*χ^2^*(2, *N* = 768) = 65.72, *P* < 0.001), self-harm (*χ^2^*(2, *N* = 768) = 44.83, *P* < 0.001) and attempted suicide (*χ^2^*(2, *N* = 768) = 37.21, *P* < 0.001).

Comparing samples at time points 1 and 2, the group differences between time to complete the survey (*t*(766) = 0.87, *P* = 0.19), gender (Fisher's exact test *P* = 0.78), employment (Fisher's exact test *P* = 0.98), relationship status (Fisher's exact test *P* = 0.12), country of residence (Fisher's exact test *P* = 0.10) and having received a formal personality disorder diagnosis (*χ^2^*(1, *N* = 768) = 0.43, *P* = 0.84) were all non-significant. Group differences regarding ethnicity were non-significant (Fisher's exact test *P* = 0.052). Regarding mental health, differences in thoughts of suicide were significant (Fisher's exact test *P* = 0.02) and self-harm were non-significant (Fisher's exact test *P* = 0.058). Differences between groups regarding attempted suicide was non-significant.

### EFA

EFA was undertaken on the 31 items of the PES with the exploratory sample (*n* = 384). The KMO value was 0.96, which was above the recommended 0.6 level,^23^ and Bartlett's test of sphericity was significant (*χ^2^*(465) = 8933.84, *P* < 0.001), indicating adequate sampling.

The results identified that a two-factor solution was optimal for the data (Supplementary Appendix 2). Factor 1 explained 27% of the variance, with factor 2 explaining an additional 27%.

These results showed considerable cross-loading of items on the two factors. Upon inspection, factor 1 was identified as relating to items describing nothingness, whereas factor 2 related to experiences of detachment. Cross-loading items were reviewed. Item 7 (‘Felt that anything you do is pointless’) and item 30 (‘Lacked a sense of direction in life’) were assigned to factor 1 based on the loadings >0.5 and relevance of these items to others loading on the factor. Item 4 (‘Felt that you do not know your place in the world’), item 12 (‘Feeling empty inside e.g. feeling like an empty shell’), item 16 (‘Having a sense of inner void that cannot be filled’) item 25 (‘Feeling somehow detached from reality, that you are not fully part of the world’) and item 26 (‘Feeling that you exist but are not really alive’) were assigned to factor 2 because of the higher factor loadings and relevance to the factor. Item 5 (‘Felt lacking in meaning and purpose in life’) and item 20 (‘Not looking forward to anything’) were removed because of loadings around 0.5 across both factors. Item 8 (‘Feeling like being stuck in a bubble, watching the world going on without you’) was removed because of cross-loading in content not clearly being relevant to either factor. Item 29 (‘Sensing that the world is bright and shiny but that you cannot be a part of it’) loaded poorly on both factors (<0.3) and so was removed. Having removed items 5, 8, 20 and 29, and re-running the EFA, factor 1 and factor 2 continued to each explain 27% of the variance.

Cronbach's alpha coefficient identified that the internal consistency of the 27 items was *α* = 0.96 (95% BCa CI 0.96–0.97). Therefore, combined with the presence of cross-loading, it was concluded that there was significant item redundancy within the measure.

### IRT

IRT was undertaken separately for the two identified factors. At this stage, factor 1 contained 13 items and factor 2 contained 14 items.

#### Factor 1: nothingness

Category characteristic curves for each item were reviewed. IIFs (see Supplementary Appendix 3) showed that items 2, 6, 9, 10, 18 and 24 provided limited information (<1) and were removed. The seven remining items of factor 1 showed discrimination ranging from 2.05 to 5.20 (Supplementary Appendix 4).

#### Factor 2: detachment

IFFs (see Supplementary Appendix 5) indicated that items 4 and 23 provided the least information (<1) and were removed. The remaining 12 items of factor 2 showed discrimination ranging from 1.96 to 3.03 (Supplementary Appendix 6).

### Face validity

**Fig. 2:**
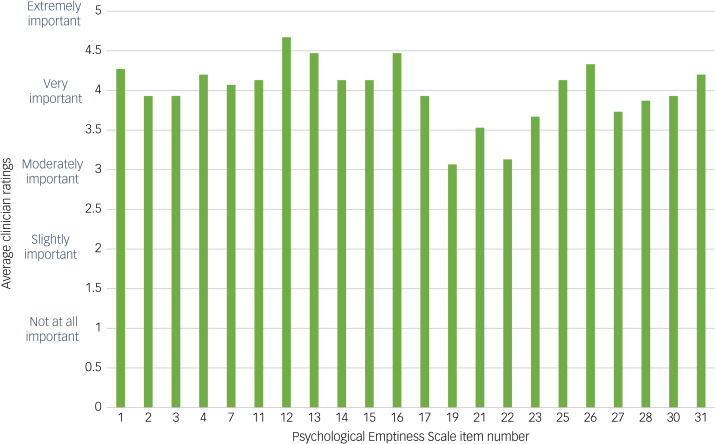
Expert clinician average ratings of scale items following item response theory.

Following IRT, decisions regarding the retention or removal of items were further informed by ratings from expert clinicians. Details relating to the clinician's experience level were gathered (Supplementary Appendix 7). Average ratings for each item of the PES were calculated (Fig. 2).

Clinician feedback indicated that all items were considered to be at least ‘moderately important’, with average scores on all items above 3. Two items received an average rating of below 3.5 (item 19 ‘Felt incapable of doing anything right’ and item 22 ‘Felt that you are just a burden to other people’). Upon review, these items were deemed to provide psychometrically important information given previous analysis, and thus were retained despite relatively lower ratings from clinicians. No further items were removed based on clinician ratings alone.

### Summary

Having initially begun with 31 items, EFA identified these to load onto two factors: factor 1, nothingness, and factor 2, detachment. These were reduced based on EFA, IRT and clinician ratings, to leave 7 and 12 items, respectively, for the two factors.

### CFA

CFA was undertaken with the confirmatory sample group data (*n* = 384), to determine whether the factor structure identified in EFA was a robust model in the shortened 19-item version of the scale.

The CFI was 0.91, and over 0.9 has been shown to indicate adequate model fit.^24^ The root mean square error of approximation was 0.09 (95% CI 0.08–0.10), indicative of a moderate fit.^21^ Factor loadings can be found in Supplementary Appendix 8. Covariance between the two factors was 0.79 (*P* < 0.001; 95% BCa CI 0.75–0.84; s.e. 0.02 and *z*-value 34.85). Following CFA, a final version of the 19-item PES was produced (Supplementary Appendix 9).

### Reliability

Following the assertion that the 19-item PES represented a suitably robust measure of emptiness with two factor subscales, assessments of reliability and validity of this short version were undertaken. Using the full sample (*N* = 768), Cronbach's alpha coefficient identified that the internal consistency of the 19-item PES was good, at *α* = 0.95 (95% BCa CI 0.95–0.96).

### Test–retest reliability

All participants who agreed to complete the original PES at a later time point had a minimum of 3 weeks between completing the measure at time point 1 and time point 2. The test–retest reliability of the 19-item measure was strong, with Spearman's *r* = 0.87 and *P* < 0.001 (95% BCa CI 0.83–0.91). Intraclass correlation coefficient estimates and their 95% confident intervals were calculated and indicated very good reliability, at 0.87 (95% BCa CI 0.82–0.90).

### Validity

Having evaluated the 19-item PES, analyses were undertaken to determine the relationship between emptiness scores and other variables of interest.

#### Sensitivity

Mean scores for the sample groups were calculated (Table 3). People who were in the high personality disorder group reported higher emptiness scores compared with the low personality disorder group. The mean scores on PES for the low personality disorder group was 19.14, and the mean PES score for the high personality disorder group was 32.07. A Mann–Whitney *U*-test identified that the difference between these scores was significant (*U*(*n*_low_ = 278, *n*_high_ = 490) = 32242.50, *P* < 0.001), and the effect size was moderate (*r* = 0.46).

**Table 3:** Mean scores by group

	*n*	Mean PES (s.d.)	Mean SAPAS (s.d.)	Mean CORE-10 (s.d.)	Mean Satisfaction with Life Scale (s.d.)	Mean Revised UCLA Loneliness Scale (s.d.)
Full sample	768	27.39 (14.08)	4.15 (1.77)	18.23 (8.34)	16.56 (7.82)	52.349 (13.02)
Low personality disorder group	278	19.14 (12.56)	2.30 (0.81)	13.80 (7.19)	20.40 (7.85)	43.20 (11.73)
High personality disorder group	490	32.07 (12.69)	5.24 (1.15)	20.74 (7.91)	14.39 (6.92)	57.54 (10.65)

PES, Psychological Emptiness Scale; SAPAS, Standardised Assessment of Personality: Abbreviated Scale; CORE-10, Clinical Outcomes in Routine Evaluation 10.

#### Convergent validity

Correlations between variables of interest were undertaken first using the full sample (*N* = 768) (Table 4), then repeated for the low personality disorder (*n* = 278) and high personality disorder (*n* = 490) groups (see Supplementary Appendix 10).

**Table 4:** Correlations between total emptiness scores, emptiness factor 1 and emptiness factor 2, and variables of interest for the full sample (*N* = 768)

Variable		Emptiness total	Emptiness factor 1	Emptiness factor 2
CORE-10 total	Spearman's rho	0.758***	0.740***	0.739***
	Upper 95% CI	0.787	0.770	0.770
	Lower 95% CI	0.726	0.706	0.705
SAPAS total	Spearman's rho	0.501***	0.486***	0.492***
	Upper 95% CI	0.552	0.538	0.543
	Lower 95% CI	0.446	0.430	0.436
Revised UCLA Loneliness Scale total	Spearman's rho	0.731***	0.695***	0.725***
	Upper 95% CI	0.763	0.730	0.757
	Lower 95% CI	0.696	0.656	0.689
Satisfaction with Life Scale total	Spearman's rho	−0.644***	−0.643***	−0.621***
	Upper 95% CI	−0.601	−0.600	−0.575
	Lower 95% CI	−0.684	−0.683	−0.662

CORE-10, Clinical Outcomes in Routine Evaluation 10; SAPAS, Standardised Assessment of Personality: Abbreviated Scale.

*** *P* < 0.001.

Regarding the whole sample, all correlations were statistically significant, with analyses identifying a strong positive relationship between total emptiness scores and psychological distress, as measured using the CORE-10 (Spearman *r*(766) = 0.76, *P* < 0.001), and loneliness (*r*(766) = 0.73, *P* < 0.001). A strong negative relationship was also identified between total emptiness and satisfaction with life (*r*(766) = –0.64, *P* < 0.001). Finally, a moderate positive relationship was identified between total emptiness scores and personality disorder traits (*r(*766) = 0.50, *P* < 0.001).

Regarding the low personality disorder group, there were strong positive relationships between total emptiness scores and scores on the CORE-10 and loneliness, as well as strong negative correlations with satisfaction with life. For the high personality disorder group, correlations revealed a strong positive relationship between total emptiness and scores on the CORE-10 and loneliness, as well as a moderate negative relationship between total emptiness and satisfaction with life. All correlations were statistically significant.

Regarding the results for the two separate factor subscales, results for the full sample were very similar to those for the total emptiness scores, and differences in relationships between the variables of interest across the two subscales were minor. Of note, factor 2 had a stronger positive relationship to loneliness than factor 1 for the full sample, high personality disorder sample and low personality disorder sample.

Correlations were undertaken to investigate the relationship between emptiness and self-harm, thoughts of suicide and attempted suicide. These correlations were undertaken using the total emptiness scores, as well as separately for the scores on the two factors identified (Table 5). This identified positive, moderately sized relationships between total emptiness scores and self-harm (*r*(758) = 0.31, *P* < 0.001), thoughts of suicide (*r*(748) = 0.39, *P* < 0.001) and attempted suicide (*r*(750) = 0.31, *P* < 0.001). Factor 1 was mostly strongly related to thoughts of suicide (*r*(768) = 0.40, *P* < 0.001). Factor 2 showed marginally stronger relationships to self-harm (*r*(768) = 0.31, *P* < 0.001) and attempted suicide (*r*(768) = 0.31, *P* < 0.001) than factor 1.

**Table 5:** Correlations between emptiness total, emptiness factor 1 and emptiness factor 2, self-harm, suicidal thoughts and suicide attempts for the full sample (*N* = 768)

		Emptiness total	Emptiness factor 1	Emptiness factor 2
Self-harm	Spearman's rho	0.311***	0.290***	0.314***
	Upper 95% CI	0.374	0.353	0.377
	Lower 95% CI	0.246	0.223	0.249
Thoughts of suicide	Spearman's rho	0.393***	0.398***	0.376***
	Upper 95% CI	0.452	0.456	0.435
	Lower 95% CI	0.332	0.337	0.314
Attempted suicide	Spearman's rho	0.312***	0.293***	0.312***
	Upper 95% CI	0.374	0.356	0.374
	Lower 95% CI	0.247	0.227	0.246

*** *P* < 0.001.

## Discussion

This study presented a robust psychometric evaluation of a new measure of emptiness based on the description of the phenomenon obtained from people with lived experience of emptiness. Beginning with 31 items, statistical analyses led to the 19-item PES demonstrating good face, convergent and temporal validity, as well as good internal reliability. The PES includes two substantially correlated yet psychometrically independent subscales. The first subscale concerns the feeling that one has no direction in life and has nothing inside, thereby merely existing (nothingness), whereas the second subscale highlights a sense of numbness, disconnectedness, indifference and lack of efficacy (detachment).

Total scores on the 19-item PES were positively correlated with loneliness, personality disorder traits, self-harm, suicidal thoughts and attempted suicide, and negatively correlated with life satisfaction in all groups, including those with and without personality disorder traits. These relationships were also the case when considering the two factors separately. This finding supports the hypotheses of multiple authors that emptiness is related to a host of psychological difficulties.^2–4^

The PES identified statistically significant differences between the low and high personality disorder groups in regard to emptiness scores. Those in the high personality disorder group reported greater emptiness scores, as would be expected because emptiness is one of the diagnostic criteria for BPD. However, the sample included individuals with a wide range of personality disorder diagnoses, and the SAPAS used to categorise participants relates to all personality disorder diagnoses.^25^ This finding suggests the possible relevance of emptiness to more than just BPD.

### Clinical implications

The work presented here has a number of implications. First, the PES may now be used as a psychometrically valid and reliable measure of emptiness within a clinical and research context. There already exists a valid instrument measuring emptiness, which is the short, five-item, single-factor Subjective Emptiness Scale.^10^ However, the PES can be an excellent alternative when a more nuanced, multifactorial and encompassing instrument is needed; for instance, with patients for whom emptiness appears to be a prominent feature. Further evaluation of the psychometric properties of the PES against other measures of emptiness would further allow researchers and clinicians to determine the most appropriate measure for their purposes and contexts.

Second, as already emphasised, this work confirmed the link between emptiness and suicidality.^3,26,27^ However, at present, emptiness is not considered within any models of suicidality. Therefore, clinicians are likely to be less attentive to the role of emptiness in risk of self-harm and suicide. An implication of the work presented here is that emptiness should be considered a significant indicator of risk. Future work should seek to explore the relationship between emptiness and risk, in comparison with previously established risk factors such as burdensomeness and belongingness. Incorporating questions related to emptiness, as well as using measures such as the PES in a clinical setting, may increase clinicians’ ability to accurately assess individuals’ level of risk to self, and prevent harm.

In evaluating the PES, items that related to lack of emotions were seen as important psychometrically, and by expert clinicians. The centrality of a lack of emotional experience in emptiness reinforces a possible defining difference between emptiness and depression, which involves the experience of negative emotions such as sadness, hopelessness, despair and guilt.^4,28^ Full awareness of this distinction may be helpful for clinicians when making sense of a person's presenting difficulties, and it may have implications for formulation, diagnosis and treatment. Further exploration of this distinction and what this may mean for treatment is called for.

### Limitations

This work has many strengths, including the thorough psychometric analyses, but a number of limitations must also be acknowledged. First, the use of an online survey may have increased the capacity to achieve a diverse sample,^29^ yet also poses limitations. The use of an online survey platform discriminates against those who are unable to access the internet, including those experiencing technological poverty, which accounts for around 10% of the population in the UK.^30^ The delivery of the project in English meant that those who do not speak English, or who face difficulties reading because of their education level, intellectual disability or other physical health condition may have been unable to take part. These barriers to participation are likely to have been greater for individuals from disadvantaged and marginalised communities, including those facing poverty, social isolation and lack of access to resources,^31^ and are likely a factor as to why this study achieved a predominantly White European sample. These difficulties in recruitment are challenging to overcome, yet common across many research methodologies, and would require assertive outreach in future studies to include excluded communities who are likely to experience emptiness.

Having been developed by an entirely White European research team at a British academic establishment, this measure will inevitably contain cultural and racial biases. Consultation was sought regarding questions related to gender; however, engaging with researchers and experts by experience, from a variety of backgrounds and perspectives would undoubtedly enable the improvement of this work to identify and rectify biases.

Thus, further validation of the PES should be undertaken using a wider variety of participants. This should include individuals from a diverse range of cultural and geographic backgrounds, including appropriately translated versions, to ensure that the PES is cross-culturally appropriate, acceptable and accessible for individuals from a wide range of sociodemographic backgrounds.^32,33^

A further limitation was the exclusion of individuals reporting a diagnosis of severe mental illness with psychotic features, as to the online nature of the study meant that we could not safeguard all participants. We acknowledge emptiness is common in this group, and therefore further research should further validate this measure for these individuals in appropriate settings, where the safety of such participants can be better ensured. Ongoing validation and work to improve the PES for these groups is required.

This may be alongside further work to prune the scale items. The decision to retain the 19 items was reached following careful consideration of all psychometric, statistical and clinically relevant data. However, the resulting Cronbach's alpha of 0.95, although considered good, is high, and may well indicate residual redundancy of items. However, at this stage these authors are confident in the balance achieved in attending to both psychometric and clinical feedback regarding the final items.

This study took place during the global COVID-19 pandemic. The associated lockdowns and resulting social and economic consequences of this pandemic had significant implications for our lives, including psychological and emotional distress. Therefore, at least in principle, the timing could be seen as a limitation of the study. However, we believe that there is no concrete reason to suggest that the nature of emptiness itself, or the scale's capacity to measure the phenomenon, could be expected to be shaped by the pandemic. Nonetheless, to ensure this, further psychometric evaluation of the measure should be undertaken as the impact of COVID-19 lessens.

In conclusion, this study presents the psychometric evaluation of the 19-item PES, demonstrating good internal consistency, robust psychometric validity and reliability. This measure's strengths lie in its brief length and transdiagnostic underpinning, while remaining true to the complexity and nuance found in self-reports of emptiness. This measure lays the foundation for considerable future research in this area. The PES has the potential to enable vital and long-awaited research into the causes, correlates, trajectory and treatment of emptiness to be undertaken. This research is necessary and vital, from both a theoretical and conceptual perspective, and significantly contributes to the aim of alleviating the immense distress felt in the form of emptiness.

## Supporting information

Herron et al. supplementary material Herron et al. supplementary material

## Data Availability

The data that support the findings of this study are available from the corresponding author, S.J.H., upon reasonable request.
