# Supplementary material for: The Psychological Emptiness Scale: a psychometric evaluation
Source: BJPsych Open. 2024 Feb 1;10(2):e42. doi: 10.1192/bjo.2023.649 (PMC10897692; doi:10.1192/bjo.2023.649)
Supplement: Herron et al. supplementary material [file S205647242300649Xsup001.docx]

Supplementary materials to:

**Psychological Emptiness Scale: A Psychometric Evaluation**

*Shona Joyce Herron, Rob Saunders, Fabio Sani & Janet Feigenbaum*

Contents

Appendix A: Floor and Ceiling Effects for PES Items (Page 2)

Appendix B: Factor Loadings for the Psychological Emptiness Scale (Page 3)

Appendix C: Item Information Functions For Factor 1 (Nothingness) (Page 4)

Appendix D: Graded Response Model related to 10-items of Factor 1 (Nothingness) (Page 4)

Appendix E: Items Information Functions for Factor 2 (Detachment) (Page 5)

Appendix F: Graded Response Model related to 10-items of Factor 2 (Detachment) (Page 5)

Appendix G: Demographic Information Regarding Expert Consult Group (N=15) (Page 7)

Appendix H: Confirmatory Factor Analysis Factor Loadings (Page 8)

Appendix I: Final Version of The Psychological Emptiness Sale (PES) (Page 9)

Appendix J: Correlations between Emptiness Scores and Variable of Interest for Low PD and High PD Samples (Page 10)

**Appendix A: Floor and Ceiling Effects for PES Items**

| PES Item No | N Floor | % Total N  (768) | N Ceiling | % Total N  (768) |
| --- | --- | --- | --- | --- |
| 1 | 167 | 21.7 | 61 | 7.9 |
| 2 | 103 | 13.4 | 98 | 12.8 |
| 3 | 35 | 4.6 | 123 | 16.0 |
| 4 | 104 | 13.5 | 204 | 26.6 |
| 5 | 97 | 12.6 | 251 | 32.7 |
| 6 | 335 | 43.6 | 50 | 6.5 |
| 7 | 117 | 15.2 | 155 | 20.2 |
| 8 | 134 | 17.4 | 174 | 22.7 |
| 9 | 152 | 19.8 | 140 | 18.2 |
| 10 | 135 | 17.6 | 97 | 12.6 |
| 11 | 227 | 29.6 | 138 | 18.0 |
| 12 | 116 | 15.1 | 139 | 18.1 |
| 13 | 118 | 15.4 | 117 | 15.2 |
| 14 | 172 | 22.4 | 185 | 24.1 |
| 15 | 59 | 7.7 | 186 | 24.2 |
| 16 | 122 | 15.9 | 185 | 24.1 |
| 17 | 131 | 17.1 | 133 | 17.3 |
| 18 | 171 | 22.3 | 171 | 22.3 |
| 19 | 159 | 20.7 | 134 | 17.4 |
| 20 | 125 | 16.3 | 169 | 22.0 |
| 21 | 220 | 28.6 | 96 | 12.5 |
| 22 | 188 | 24.5 | 154 | 20.1 |
| 23 | 113 | 14.7 | 133 | 17.3 |
| 24 | 68 | 8.9 | 266 | 34.6 |
| 25 | 155 | 20.2 | 154 | 20.1 |
| 26 | 158 | 20.6 | 198 | 25.8 |
| 27 | 256 | 33.3 | 115 | 15.0 |
| 28 | 278 | 36.2 | 133 | 17.3 |
| 29 | 359 | 46.7 | 61 | 7.9 |
| 30 | 130 | 16.9 | 221 | 28.8 |
| 31 | 125 | 16.3 | 138 | 18.0 |

**Appendix B: Factor Loadings for the Psychological Emptiness Scale**

|  | | **Factor 1** | | **Factor 2** | | **Uniqueness** | |
| --- | --- | --- | --- | --- | --- | --- | --- |
| Emptiness 1 |  |  |  | 0.749 |  | 0.399 |  |
| Emptiness 2 |  | 0.618 |  |  |  | 0.487 |  |
| Emptiness 3 |  |  |  | 0.750 |  | 0.373 |  |
| Emptiness 4 |  | 0.670 |  | 0.417 |  | 0.378 |  |
| Emptiness 5 |  | 0.601 |  | 0.517 |  | 0.371 |  |
| Emptiness 6 |  | 0.555 |  |  |  | 0.608 |  |
| Emptiness 7 |  | 0.654 |  | 0.456 |  | 0.365 |  |
| Emptiness 8 |  | 0.447 |  | 0.555 |  | 0.491 |  |
| Emptiness 9 |  | 0.545 |  |  |  | 0.597 |  |
| Emptiness 10 |  | 0.409 |  |  |  | 0.781 |  |
| Emptiness 11 |  | 0.726 |  |  |  | 0.342 |  |
| Emptiness 12 |  | 0.432 |  | 0.686 |  | 0.343 |  |
| Emptiness 13 |  |  |  | 0.780 |  | 0.339 |  |
| Emptiness 14 |  |  |  | 0.686 |  | 0.390 |  |
| Emptiness 15 |  |  |  | 0.622 |  | 0.511 |  |
| Emptiness 16 |  | 0.442 |  | 0.627 |  | 0.411 |  |
| Emptiness 17 |  |  |  | 0.619 |  | 0.475 |  |
| Emptiness 18 |  | 0.541 |  |  |  | 0.587 |  |
| Emptiness 19 |  | 0.759 |  |  |  | 0.396 |  |
| Emptiness 20 |  | 0.578 |  | 0.498 |  | 0.418 |  |
| Emptiness 21 |  | 0.661 |  |  |  | 0.461 |  |
| Emptiness 22 |  | 0.776 |  |  |  | 0.357 |  |
| Emptiness 23 |  |  |  | 0.611 |  | 0.544 |  |
| Emptiness 24 |  | 0.527 |  |  |  | 0.605 |  |
| Emptiness 25 |  | 0.411 |  | 0.665 |  | 0.389 |  |
| Emptiness 26 |  | 0.449 |  | 0.638 |  | 0.391 |  |
| Emptiness 27 |  |  |  | 0.562 |  | 0.551 |  |
| Emptiness 28 |  | 0.823 |  |  |  | 0.255 |  |
| Emptiness 29 |  | 0.274 |  | 0.230 |  | 0.777 |  |
| Emptiness 30 |  | 0.609 |  | 0.425 |  | 0.449 |  |
| Emptiness 31 |  |  |  | 0.720 |  | 0.382 |  |
|  | | | | | | | |
| NB.  Applied rotation method is varimax. | | | | | | | |

**Appendix C: Item Information Functions For Factor 1 (Nothingness)**

**Appendix D: Graded Response Model related to 10-items of Factor 1 (Nothingness)**

| **Table 4**  *Graded Response Model related to 10-items of Factor 1 (N= 384)* | | | | | | |
| --- | --- | --- | --- | --- | --- | --- |
| Log likelihood = -2784.00 | | | | | | |
|  | **Coefficient** | **Standard Error** | **z** | **P>\|z\|** | **[95% conf. interval]** | |
| **Emptiness7** |  |  |  |  |  |  |
| Discrim | 2.42 | .22 | 11.00 | .00 | 1.99 | 2.85 |
| **Emptiness11** |  |  |  |  |  |  |
| Discrim | 3.25 | .30 | 10.76 | .00 | 2.66 | 3.85 |
| **Emptiness19** |  |  |  |  |  |  |
| Discrim | 2.43 | .22 | 11.06 | .00 | 2.00 | 2.86 |
| **Emptiness21** |  |  |  |  |  |  |
| Discrim | 2.40 | .22 | 11.07 | .00 | 1.98 | 2.82 |
| **Emptiness22** |  |  |  |  |  |  |
| Discrim | 2.75 | .25 | 11.00 | .00 | 2.26 | 3.24 |
| **Emptiness28** |  |  |  |  |  |  |
| Discrim | 5.20 | .62 | 8.41 | .00 | 3.99 | 6.41 |
| **Emptiness30** |  |  |  |  |  |  |
| Discrim | 2.05 | .189 | 10.86 | .00 | 1.68 | 2.42 |

**Appendix E: Items Information Functions for Factor 2 (Detachment)**

**Appendix F: Graded Response Model related to 10-items of Factor 2 (Detachment)**

| Log likelihood = -4609.90 | | | | | | |
| --- | --- | --- | --- | --- | --- | --- |
|  | **Coefficient** | **Std. err.** | **z** | **P>\|z\|** | **[95% conf. interval]** | |
| **Emptiness 1** |  |  |  |  |  |  |
| Discrim | 2.72 | .24 | 11.12 | .00 | 2.24 | 3.20 |
| **Emptiness 3** |  |  |  |  |  |  |
| Discrim | 2.76 | .24 | 11.33 | .00 | 2.28 | 3.24 |
| **Emptiness 12** |  |  |  |  |  |  |
| Discrim | 3.02 | .26 | 11.53 | .00 | 2.51 | 3.24 |
| **Emptiness 13** |  |  |  |  |  |  |
| Discrim | 3.03 | .27 | 11.43 | .00 | 2.51 | 3.55 |
| **Emptiness 14** |  |  |  |  |  |  |
| Discrim | 2.75 | .24 | 11.39 | .00 | 2.28 | 3.23 |
| **Emptiness 15** |  |  |  |  |  |  |
| Discrim | 2.02 | .18 | 11.23 | .00 | 1.70 | 2.41 |
| **Emptiness 16** |  |  |  |  |  |  |
| Discrim | 2.46 | .21 | 11.55 | .00 | 2.04 | 2.88 |
| **Emptiness 17** |  |  |  |  |  |  |
| Discrim | 2.16 | .19 | 11.38 | .00 | 1.78 | 2.53 |
| **Emptiness 25** |  |  |  |  |  |  |
| Discrim | 2.67 | .23 | 11.37 | .00 | 2.21 | 3.13 |
| **Emptiness 26** |  |  |  |  |  |  |
| Discrim | 2.49 | .22 | 11.44 | .00 | 2.60 | 2.91 |
| **Emptiness 27** |  |  |  |  |  |  |
| Discrim | 1.96 | .18 | 10.76 | .00 | 1.60 | 2.31 |
| **Emptiness 31** |  |  |  |  |  |  |
| Discrim | 2.74 | .24 | 11.56 | .00 | 2.27 | 3.20 |

**Appendix G: Demographic Information Regarding Expert Consult Group (N=15)**

| **Current Clinical Role** | **N (%)** |
| --- | --- |
| Clinical Psychologist | 3 (20.0) |
| Counselling Psychologist | 2 (13.3) |
| CBT Therapist | 1 (6.7) |
| Psychotherapist | 2 (13.3) |
| Psychological Therapist | 1 (6.7) |
| Assistant Psychologist | 1 (6.7) |
| Trainee Clinical Psychologist | 5 (33.3) |
| **Number of Years Working with Clients Experiencing Emptiness** |  |
| <1 year | 4 (26.7) |
| 1-2 years | 0 (0.0) |
| 3-4 years | 3 (20.0) |
| 5-6 years | 0 (0.0) |
| 7-8 years | 3 (20.0) |
| 9-10 years+ | 5 (33.3) |
| **Most Common Diagnoses of Clients Worked With** |  |
| Depression including Chronic Depression | 11 (73.3) |
| Borderline Personality Disorder | 9 (60.0) |
| Emotionally Unstable Personality Disorder | 4 (26.7) |
| Bipolar Disorder | 3 (20.0) |
| Avoidant Personality Disorder | 2 (13.3) |
| Personality Disorders (Non-Specified) | 2 (13.3) |
| PTSD/Trauma | 2 (13.3) |
| Substance Use Disorders | 2 (13.3) |
| Eating Disorders | 2 (13.3) |
| Anxiety | 2 (13.3) |
| Obsessive Compulsive Disorder | 1 (6.7) |
| Generalised Anxiety Disorder | 1 (6.7) |
| Dysthymia | 1 (6.7) |
| Autism Spectrum Disorder | 1 (6.7) |

**Appendix H:** **Confirmatory Factor Analysis Factor Loadings**

|  | | | | | | | | | | | **95% Confidence Interval** | | | | | |
| --- | --- | --- | --- | --- | --- | --- | --- | --- | --- | --- | --- | --- | --- | --- | --- | --- |
| **Factor** | **Indicator** | **Estimate** | | **Std. Error** | | **z-value** | | ***p*** | | | **Lower** | | | | **Upper** | |
| **Factor 1** | Emptiness 7 |  | 0.807 |  | 0.042 |  | 19.034 | |  | < .001 | |  | 0.724 |  | | 0.890 |
|  | Emptiness 11 |  | 0.910 |  | 0.046 |  | 19.827 | |  | < .001 | |  | 0.820 |  | | 1.000 |
|  | Emptiness 19 |  | 0.807 |  | 0.045 |  | 18.087 | |  | < .001 | |  | 0.719 |  | | 0.894 |
|  | Emptiness 21 |  | 0.746 |  | 0.044 |  | 16.855 | |  | < .001 | |  | 0.659 |  | | 0.833 |
|  | Emptiness 22 |  | 0.806 |  | 0.048 |  | 16.957 | |  | < .001 | |  | 0.713 |  | | 0.899 |
|  | Emptiness 28 |  | 0.939 |  | 0.047 |  | 19.983 | |  | < .001 | |  | 0.847 |  | | 1.031 |
|  | Emptiness 30 |  | 0.716 |  | 0.049 |  | 14.608 | |  | < .001 | |  | 0.620 |  | | 0.813 |
| **Factor 2** | Emptiness 1 |  | 0.652 |  | 0.039 |  | 16.844 | |  | < .001 | |  | 0.576 |  | | 0.728 |
|  | Emptiness 3 |  | 0.636 |  | 0.040 |  | 15.857 | |  | < .001 | |  | 0.558 |  | | 0.715 |
|  | Emptiness 12 |  | 0.772 |  | 0.040 |  | 19.528 | |  | < .001 | |  | 0.695 |  | | 0.849 |
|  | Emptiness 13 |  | 0.708 |  | 0.041 |  | 17.238 | |  | < .001 | |  | 0.627 |  | | 0.788 |
|  | Emptiness 14 |  | 0.847 |  | 0.047 |  | 17.893 | |  | < .001 | |  | 0.754 |  | | 0.939 |
|  | Emptiness 15 |  | 0.659 |  | 0.042 |  | 15.812 | |  | < .001 | |  | 0.577 |  | | 0.740 |
|  | Emptiness 16 |  | 0.833 |  | 0.045 |  | 18.681 | |  | < .001 | |  | 0.746 |  | | 0.920 |
|  | Emptiness 17 |  | 0.697 |  | 0.044 |  | 15.761 | |  | < .001 | |  | 0.610 |  | | 0.783 |
|  | Emptiness 25 |  | 0.770 |  | 0.045 |  | 16.966 | |  | < .001 | |  | 0.681 |  | | 0.859 |
|  | Emptiness 26 |  | 0.828 |  | 0.047 |  | 17.637 | |  | < .001 | |  | 0.736 |  | | 0.920 |
|  | Emptiness 27 |  | 0.706 |  | 0.048 |  | 14.612 | |  | < .001 | |  | 0.612 |  | | 0.801 |
|  | Emptiness 31 |  | 0.744 |  | 0.041 |  | 17.985 | |  | < .001 | |  | 0.663 |  | | 0.825 |

**Appendix I: Final Version of The Psychological Emptiness Sale (PES)**

| **Please specify how often you have had the following experiences over the last month:** | | **Never**  **0** | **Sometimes**  **1** | **Often**  **2** | **All the Time**  **3** |
| --- | --- | --- | --- | --- | --- |
| 1 | Felt unable to feel emotions (e.g., joy, happiness, anger, sadness) |  |  |  |  |
| 2 | Felt indifferent to anything that goes on around you |  |  |  |  |
| 3 | Felt that anything that you might do is pointless |  |  |  |  |
| 4 | Felt that you are nothing |  |  |  |  |
| 5 | Felt empty inside (e.g., feeling like an “empty shell”) |  |  |  |  |
| 6 | Felt emotionally numb |  |  |  |  |
| 7 | Felt that positive things such as love or joy do not stick to you, that they just pass through you |  |  |  |  |
| 8 | Felt that you are just going through the motions |  |  |  |  |
| 9 | Had a sense of an inner void that cannot be filled |  |  |  |  |
| 10 | Had neither desires nor motivations |  |  |  |  |
| 11 | Felt incapable of doing anything right |  |  |  |  |
| 12 | Felt that you had no impact on others |  |  |  |  |
| 13 | Felt that you are just a burden to other people |  |  |  |  |
| 14 | Feeling somehow detached from reality, that you are not fully part of the world |  |  |  |  |
| 15 | Felt that you just ‘exist’, but are not really ‘alive’ |  |  |  |  |
| 16 | Felt that between you and the outside world there is some sort of barrier (e.g., a fog, a veil, a chasm or gulf) |  |  |  |  |
| 17 | Felt that you had nothing to offer the world, that you are worthless |  |  |  |  |
| 18 | Lacked a sense of direction in life |  |  |  |  |
| 19 | Were disengaged, and not really caring about anything |  |  |  |  |
|  | | | | | |

**Appendix J: Correlations between Emptiness Scores and Variable of Interest for Low PD and High PD Samples**

| *Correlations between Total Emptiness Scores, Emptiness Factor 1 and Emptiness Factor 2, and variables of interest for Low PD Sample (N=278)* | | | | |
| --- | --- | --- | --- | --- |
| **Variable** |  | **Emptiness Total** | **Emptiness Factor 1** | **Emptiness Factor 2** |
| **CORE-10 Total** | Spearman's rho | 0.735 *** | 0.721 *** | 0.707 *** |
|  | Upper 95% CI | 0.785 | 0.773 | 0.761 |
|  | Lower 95% CI | 0.675 | 0.659 | 0.643 |
| **SAPAS Total** | Spearman's rho | 0.315 *** | 0.278 *** | 0.330 *** |
|  | Upper 95% CI | 0.417 | 0.383 | 0.431 |
|  | Lower 95% CI | 0.205 | 0.166 | 0.221 |
| **Loneliness Total** | Spearman's rho | 0.662 *** | 0.642 *** | 0.646 *** |
|  | Upper 95% CI | 0.723 | 0.706 | 0.710 |
|  | Lower 95% CI | 0.590 | 0.567 | 0.572 |
| **Satisfaction with Life Total** | Spearman's rho | -0.597 *** | -0.617 *** | -0.558 *** |
|  | Upper 95% CI | -0.516 | -0.538 | -0.472 |
|  | Lower 95% CI | -0.668 | -0.685 | -0.634 |
| NB. **p*< .05, ***p*< .01, ****p*< .001 | | | | |
|  | | | | |
| *Correlations between Total Emptiness Scores, Emptiness Factor 1 and Emptiness Factor 2, and variables of interest for High PD Sample (N=490)* | | | | |
| **Variable** |  | **Emptiness Total** | **Emptiness Factor 1** | **Emptiness Factor 2** |
| **CORE-10 Total** | Spearman's rho | 0.707 *** | 0.670 *** | 0.692 *** |
|  | Upper 95% CI | 0.748 | 0.716 | 0.736 |
|  | Lower 95% CI | 0.659 | 0.618 | 0.643 |
| **SAPAS Total** | Spearman's rho | 0.277 *** | 0.266 *** | 0.273 *** |
|  | Upper 95% CI | 0.357 | 0.347 | 0.353 |
|  | Lower 95% CI | 0.194 | 0.182 | 0.189 |
| **Loneliness Total** | Spearman's rho | 0.637 *** | 0.580 *** | 0.640 *** |
|  | Upper 95% CI | 0.687 | 0.636 | 0.690 |
|  | Lower 95% CI | 0.582 | 0.519 | 0.585 |
| **Satisfaction with Life Total** | Spearman's rho | -0.578 *** | -0.566 *** | -0.559 *** |
|  | Upper 95% CI | -0.516 | -0.503 | -0.495 |
|  | Lower 95% CI | -0.634 | -0.623 | -0.617 |
| NB. **p*< .05, ***p*< .01, ** *p*< .001 | | | | |
